# Microglia-Derived Interleukin 23: A Crucial Cytokine in Alzheimer's Disease?

**DOI:** 10.3389/fneur.2021.639353

**Published:** 2021-04-07

**Authors:** Louisa Nitsch, Linda Schneider, Julian Zimmermann, Marcus Müller

**Affiliations:** ^1^Department of Neurology, University Hospital Bonn, Bonn, Germany; ^2^Department of Surgery, University Hospital Bonn, Bonn, Germany

**Keywords:** interleukin 23, microglia, neuroinflammation, antibody therapy, Alzheimer's disease

## Abstract

Neuronal cell death, amyloid β plaque formation and development of neurofibrillary tangles are among the characteristics of Alzheimer's disease (AD). In addition to neurodegeneration, inflammatory processes such as activation of microglia and astrocytes are crucial in the pathogenesis and progression of AD. Cytokines are essential immune mediators of the immune response in AD. Recent data suggest a role of interleukin 23 (IL-23) and its p40 subunit in the pathogenesis of AD and corresponding animal models, in particular concerning microglia activation and amyloid β plaque formation. Moreover, in animal models, the injection of anti-p40 antibodies resulted in reduced amyloid β plaque formation and improved cognitive performance. Here, we discuss the pathomechanism of IL-23 mediated inflammation and its role in AD.

## Introduction

Alzheimer's disease (AD) is the most common form of dementia and affects millions of people worldwide (1). Despite the immense progress in AD research over the recent years, many aspects of the underlying pathomechanism remain elusive and new approaches for effective therapies are needed. Besides neurodegeneration, the disease-accelerating role of neuroinflammation has become a focus of research in AD. Since the central role of interleukin 23 (IL-23) in neuroinflammation, especially in multiple sclerosis (MS), has become clear, several interesting studies about AD and IL-23 have been published. Therefore, this review provides an overview of the current data on IL-23 mediated neuroinflammation in AD, open aspects for further research and possible therapeutic approaches.

## IL-23

Cytokines are peptide hormones that act as messengers of the immune system and modulate the immune response in an autocrine, paracrine, or endocrine manner (2). They regulate activation or inhibition of immune cells, control their differentiation, proliferation and chemotaxis. In particular, the successful application of antibodies modulating the cytokine function for the treatment of a variety of diseases has further advanced the research in the field of cytokines and cytokine-inhibiting therapies.

A subgroup of cytokines are the interleukins. IL-23 consists of a unique p19 and a common p40 subunit, which is shared by the structurally related IL-12 (3). It appears that the heterodimeric molecule is the bioactive cytokine and both subunits, p19/p40 for IL-23 and p35/p40 for IL-12 must be co-expressed in the same cell to generate the bioactive form. However, some data also show an effector function of p40 alone as discussed below. Since the first study demonstrated that IL-23 and not the structurally similar IL-12 is the central cytokine contributing to the pathogenesis of autoimmune diseases (4, 5), the importance of IL-23 in neuroinflammation has been further deciphered in many preclinical and clinical studies. IL-23 is primarily secreted by antigen presenting cells (APC) like dendritic cells, macrophages, and B cells (3, 6, 7). The local production of IL-23 in the CNS has been demonstrated for astrocytes and infiltrating macrophages under inflammatory conditions (5, 8). In addition, some studies demonstrated the secretion of the IL-12/IL-23 subunit p40 by microglia (9, 10), while others provided evidence that microglia secrete the bioactive cytokine IL-23 upon activation (11–13).

**Figure 1:**
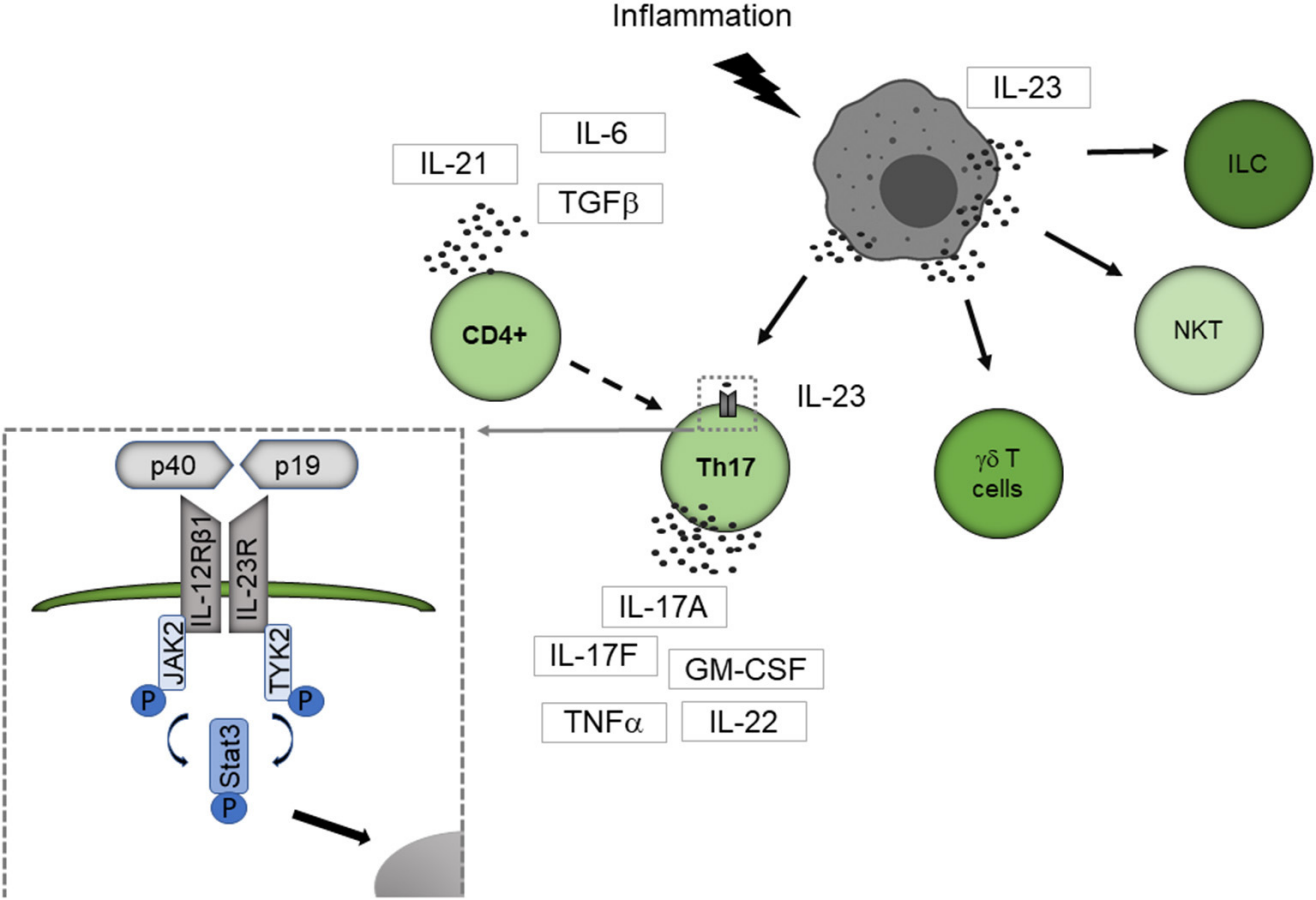
IL-23 mediated activation of the immune system. IL-23 is primarily secreted by APC like dendritic cells, macrophages, and B cells. Best known responders to IL-23 stimulation are Th17 cells, a distinct subpopulation of γδ T cells, subsets of natural killer T cells, and innate lymphoid cells. IL-23 binds to its specific receptor complex, which consists of a unique IL-23 receptor subunit and a IL12β1 subunit. γδ T cells express the IL-23 receptor constitutively, but naive CD4+ cells lack the IL-23 receptor. CD4+ cells are therefore first activated by other cytokines such as TGF-β, IL-6, or IL-21, then differentiate into Th17 cells and express the IL-23 receptor. By binding to the IL-23 receptor, IL-23 leads conformational change of the receptor that promotes the phosphorylation of JAK2 and Tyk2 leading to phosphorylation of STAT factors. Thereby STAT3 is primarily activated. The activated STAT protein enters the nucleus to exert its biological effects. ILC, innate lymphoid cell; NKT, natural killer cell.

Figure 1 summarizes how IL-23 activates the immune system. Best known responders to IL-23 stimulation are the CD4 T helper subset T helper 17 (Th17) cells, a distinct subpopulation of γδ T cells, subsets of natural killer T cells, and innate lymphoid cells (14). IL-23 binds to its specific receptor complex, which consists of a unique IL-23 receptor subunit and a IL12β1 subunit. γδ T cells express the IL-23 receptor constitutively, but naive CD4+ cells lack the IL-23 receptor. CD4+ cells are therefore first activated by other cytokines such as the transforming growth factor (TGFβ), IL-6 or IL-21, then differentiate into Th17 cells and express the IL-23 receptor (15–17).

By binding to the IL-23 receptor, IL-23 leads to a conformational change of the receptor, which promotes the phosphorylation of Janus kinase 2 (JAK2) and tyrosine kinase 2 (Tyk2) leading to phosphorylation of signal transducer and activator of transcription (STAT) factors with STAT3 primarily activated (18). The activated STAT protein enters the nucleus to exert its biological effects. This leads to the secretion of the Th17 cell characteristic cytokines IL-17A, IL-17F and several other proinflammatory cytokines like IL-22, the granulocyte-macrophage colony-stimulating factor (GM-CSF) or the tumor necrosis factor (TNFα) (14).

Among the different cell subtypes expressing the IL-23 receptor, Th17 cells could be established as key players in neuroinflammation, particularly in MS (19, 20). Beside these well-known responders of lymphocytic origin, expression of the IL-23 receptor on macrophages/monocytes, microglia, and dendritic cells was described (21, 22), enabling these cells to directly interact with IL-23. Thereby, IL-23 enhances the cytokine production of IL-23 receptor expressing myeloid cells (21, 22).

## IL-23 and Neuroinflammation

The crucial role of IL-23 in the pathogenesis of a vast variety of autoimmune diseases like inflammatory bowel diseases, rheumatoid arthritis, and psoriasis has been clearly demonstrated [(23–27), summarized in Table 1]. IL-23 is also important in the atherogenesis and progression of atherosclerotic plaques (28, 29).

**Table 1:** Role of IL-23 in the pathogenesis of different diseases.

**Disease**	**IL-23 effect**	**References**
Inflammatory bowel diseases	Pathogenic	(24, 25)
Rheumatoid arthritis	Pathogenic	(25, 27)
Psoriasis	Pathogenic	(23–26)
Atherosclerosis	Pathogenic	(28, 29)
MS	Pathogenic	(13–15, 30–32)
Stroke/postischemic inflammation	Pathogenic	(33, 34)
Infections	Protective	(35–37)

Numerous studies could demonstrate the significance of IL-23 in neuroinflammation as well. Most of the data on IL-23 and neuroinflammation have been derived from studies on the pathogenesis of MS and corresponding animal models (38). The central role of IL-23 in the development of MS is beyond doubt. IL-23 is increased in serum, cerebrospinal fluid (CSF), and lesional tissue of MS patients (13–15). Animal models emphasize the non-redundant role of IL-23 in MS, as an experimental autoimmune encephalitis cannot be induced in mice lacking IL-23 or the receptor complex (30–32). Nevertheless, there are also studies suggesting IL-23 is critical in stroke patients, especially in the postischemic inflammatory phase (33, 34). Furthermore, the IL-23 signaling pathway is part of the defense mechanism in viral, bacterial, and fungal infections of the CNS (35–37). To further investigate how IL-23 mediates neuroinflammation in different animal models, we have recently established a mouse model with astrocyte-specific expression of IL-23 revealing unexpectedly, a spontaneous B cell accumulation in the cerebellum (39).

With the increasing number of studies demonstrating the importance of IL-23 in neuroinflammation, several studies have also investigated the influence of IL-23 on AD. Therefore, this review will give an overview of the role of IL-23 in AD.

## AD and Neuroinflammation

Characteristics of AD are amyloid β (Aβ) plaques, which are extracellular deposits of Aβ derived from the β-amyloid precursor protein (APP), neurofibrillary tangles composed of hyperphosphorylated tau and neuronal cell death (40). In addition to neurodegeneration, neuroinflammation is crucial in the pathogenesis and progression of AD (40). Although it is not clear to what extent neuroinflammation contributes to the pathogenesis of AD, it is generally acknowledged that the immune system influences the disease progression.

Neuroinflammation in AD is mainly promoted by CNS-resident cells like microglia and astrocytes. Microglia are CNS-resident cells of myeloid origin with immune-modulating and phagocytic capabilities (41). Activation of the innate immune system in AD seems to follow Aβ deposition. However, studies of patients with mild cognitive impairment demonstrate neuroinflammation even in the early phase (42, 43).

Aβ plaques are surrounded by reactive astrocytes and activated microglial cells (44, 45). The role of microglia in AD initiation and progression are debated, with conflicting reports regarding their detrimental or protective function (46). Microglia and astrocytes can remove Aβ by uptake and degradation or extracellularly degrade Aβ by enzyme secretion (40). However, they also lead to increased Aβ levels and contribute to tissue reaction and destruction in AD especially during disease progression (47). In addition, activation of microglia and complement-dependent pathways mediates synapse loss in AD (48). The state of activation appears to determine whether microglia have a protective or detrimental role in AD (49). Microglial mediated neuroinflammation is increased in AD while microglial-mediated Aβ clearance mechanisms are diminished (41).

Microglia and astrocytes are the major source of proinflammatory cytokines as essential regulators of the immune response in AD (50). IL-1, IL-6, IL-12, IL-23, GM-CSF, TNF-, C-X-C motif chemokine ligand 10 (CXCL10) are detectable or upregulated in animal models of AD, in the brain or CSF from AD patients (47, 51). APP/presenilin 1 (APP/PS1) mice, a well-established mouse model to study amyloid pathology in AD, show reduced plaque burden and Aβ levels if genetically deficient for CXCR3, the receptor for CXCL10 (52). The proinflammatory cytokine milieu in the AD brain contributes directly or indirectly to neuronal damage. Aβ stimulation results in secretion of proinflammatory cytokines, which trigger neuronal hyperexcitability and synaptic dysfunction (40). Moreover, cytokines stimulate the secretion of inducible nitric oxide synthase in microglia and astrocytes, which is toxic to neurons at high concentrations (40).

## IL-23 and AD

As the relevance of IL-23 in neuroinflammation, particularly in MS, has become evident, the question of how IL-23 affects inflammatory processes in AD has arisen. Therefore, several descriptive and experimental studies addressed the impact of IL-12/23 p40 and IL-23 signaling on AD in recent years.

Single nucleotide polymorphisms in the IL-12/23 subunit p40 (rs3212227) (53) and IL-23 receptor polymorphisms are associated with AD in a northern Han Chinese population (54).

AD patients show higher peripheral levels of IL-23 (55) and the concentration of the subunit p40 was identified as a serum marker for the prediction of the Aβ load in an AD cohort (56). A plasma multianalyte profiling study of patients with mild cognitive impairment and AD demonstrated an association of plasma p40 levels with abnormal cognitive performance (57).

In contrast, a smaller study demonstrated reduced p40 concentration in CSF in patients with cognitive impairment including AD in early phases of the disease with a median Mini-Mental State Examination (MMSE) score of 23 (58). However, this study additionally found CSF IL-12/23 p40 concentrations correlated positively with CSF concentrations of Aβ1-42 and phosphorylated tau protein but not MMSE score in the total study population including patients with mild cognitive impairment, AD, and other dementia forms. But in AD patients CSF IL-12/23 p40 only correlated positively with CSF P-Tau (58). The most extensive study concerning the role of IL-23 in AD was performed by vom Berg et al. (59). They found increased expression of p40 in microglia in APP/PS1 mice and increased p40 in cerebrospinal fluid of AD patients. The cognitive performance measured with the MMSE score correlated in this study negatively with CSF IL-12/23 p40 levels. However, it should be noted that the number of AD patients studied was small (*n* = 7). Furthermore, p40 appears to contribute to the extent of cerebral plaque formation and activation of microglia in the mouse model. Cytokine-knockout (p19, p35, and p40) APP/PS1 mice showed reduced microglial activation and disease severity along with diminished accumulation of Aβ in young and older mice (59). Thereby, loss of the common IL-12/23 p40 subunit did show the greatest impact on Aβ plaque burden, whereas deficiency of the unique subunits IL-23 p19 or IL-12 p35 results in a similar but weaker reduction. Moreover, experiments with bone marrow-chimeric mice indicated that microglial cell-derived IL-12/23 p40, but not peripheral myeloid cell-derived IL-12/23 p40 is involved in the extent of in Aβ plaque load. This finding further illustrates the central role of microglia in mediating p40-related effects on Aβ burden (59). The p40 production by microglia was associated with the *de novo* expression of the activation marker CD11c indicating modulation of microglia activity when producing p40 (59). In addition, after injection of anti-p40 antibodies before or after the onset of amyloid accumulation, the mice showed reduced Aβ formation and improved cognitive performance (59). Thus, both female and male mice were used for this study and a gender bias cannot be ruled out. Another study examined the gender-specific effect in mice lacking IL12p40. Eede et al. found that IL12p40 deficiency reduces Aβ plaque burden in male APP23 mice, while female mice had a significant reduction in soluble Aβ1-40 without changes in Aβ plaque burden (60). Furthermore, plasma and brain cytokine levels are altered differently in female vs. male APP23 mice lacking IL12p40.

### Which Cells Mediate the Effects of IL-23 in AD?

**Figure 2:**
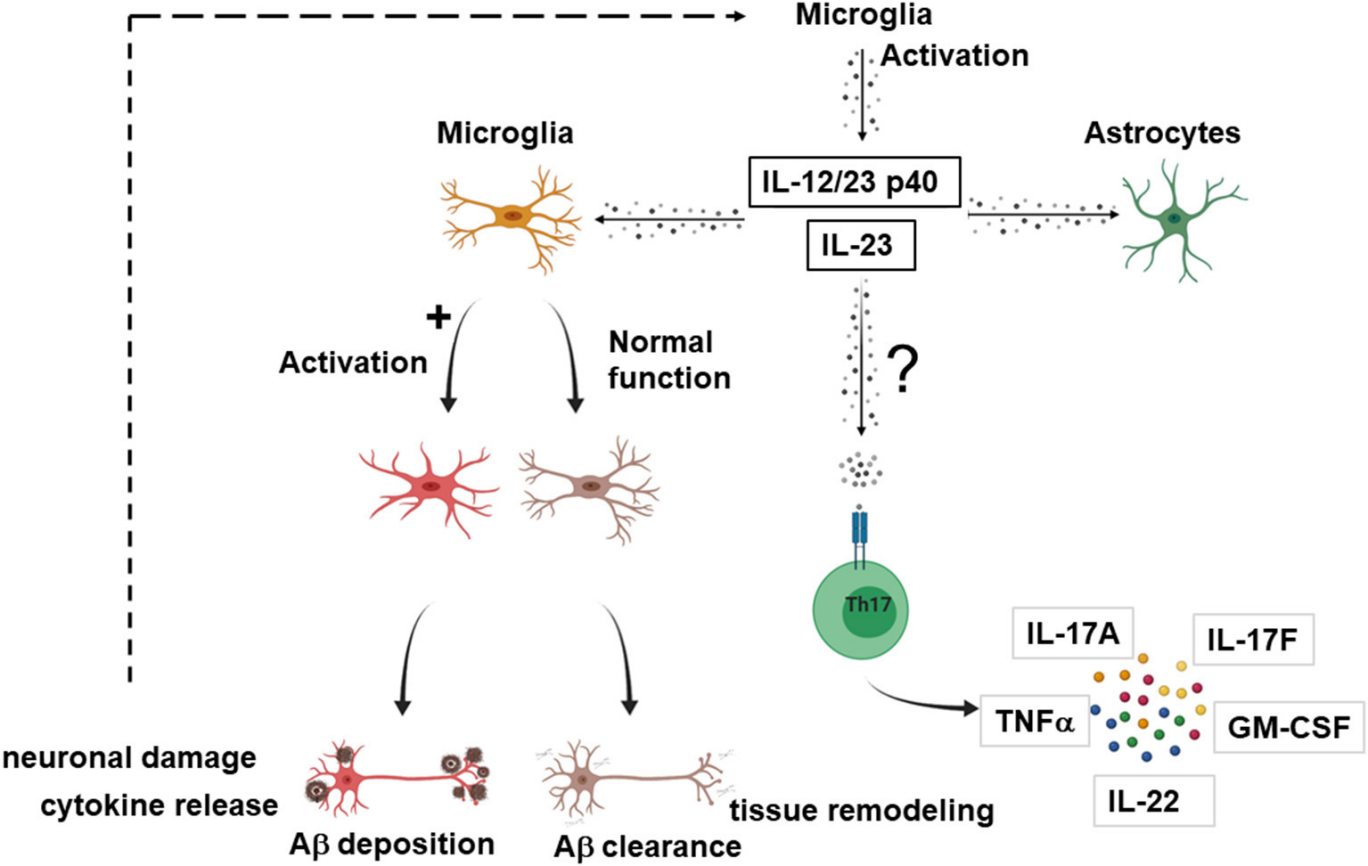
Possible effector cells in IL-23 mediated neuroinflammation in AD. Suggested model how IL-23 and IL-12/23 p40 drives neuroinflammation in AD. Upon activation, microglia secrete IL-23 or/and IL-12/23 p40. IL-23 and IL-12/23 p40 lead to a shift of microglia from Aβ clearance and tissue remodeling toward an activated state with production of proinflammatory cytokines, inhibition of the clearance of Aγδ neuronal damage, which in turn could further enhance the IL-23 secretion. IL-23 and IL-12/23 p40 could also lead to activation of astrocytes. Nevertheless, it cannot be excluded that the effector cells for IL-23 signaling are partly T cells, as described for other neuroinflammatory diseases. The figure was generated with BioRender.com.

While the number of leukocyte subpopulations known to respond to IL-23 is growing (14), the effector cells upon IL-23 signaling in the context of AD remain elusive. Although the actions of IL-23 in other neuroinflammatory processes like MS are mediated *via* Th17 cells (19, 20), in AD, IL-23 and IL-12/23 p40 might act through novel mechanisms independent from T cells. Figure 2 provides a proposed mechanism how IL-23 drives neuroinflammation in AD.

Vom Berg et al. provided a hypothesis involving cells of the innate immunity as effectors in the p40 driven signaling pathway (59). One possible scenario covers the sustained Aβ-driven release of p40 by microglia that binds to the IL12Rβ1 receptor on adjacent astrocytes in a paracrine manner. As a second scenario, autocrine activation of microglia by binding of p40 to the IL12Rβ1 receptor on microglia themselves is suggested, which promotes AD pathology.

However, it should be noted that the p40 subunit alone is in generally not considered a bioactive form. It appears that both subunits, p40 and p19 for IL-23, p40 and p35 for IL-12, must be co-expressed in the same cell to generate the bioactive form (IL-12 or IL-23). But several studies in fact show that p40 alone can act as a messenger substance (61). The p40 homodimer is capable of inducing the expression of immune factors in microglia *via* the IL-12Rβ1 (62).

Further data are needed to elucidate if neuroinflammation in AD is driven by IL-12/23 p40, IL-23 or even partly by IL-12. Since p40 is a subunit of both IL-23 and IL-12, this is difficult to determine from the data. The results of the APP/PS1 mouse model with deletion of the unique subunits IL-23 p19 or IL-12 p35, which results in a weaker but similar reduction of the plaque load, speak against the fact that the effect is mediated by p40 alone (59).

A study analyzing Aβ clearance in IL-23-treated microglia further enlightened, which effector cells might respond to p40 or IL-23. In a human macrophage cell line Aβ42 incubation increased the expression of IL-17, IL-18, IL-23, whereas the same cytokines impaired Aβ clearance by macrophages or microglia (63). The inhibitory effects of IL-18 were blocked by IL-23 or IL-17 neutralizing antibodies while the inhibitory effects of IL-23 were blocked by IL-17 neutralizing antibodies pointing to an interaction of IL-17, IL-18, and IL-23 and microglia for the Aβ clearance.

In another mouse model, the senescence-accelerated mouse prone-8 model (SAMP8), Tan et al. screened the cerebral expression of IL-12/23 in 3-, 7-, and 11-month-old mice and demonstrated that these cytokine levels in the brain were upregulated during aging (64). By *in vivo* infusion of non-viral small interfering RNA (siRNA) to knock down the common IL-12/23 subunit p40 in the brain, they demonstrated that these p40-deficient mice had significantly diminished cerebral Aβ42 levels, reduced synaptic and neuronal loss, and reversed cognitive impairments. In addition, treatment of the SAMP8 mice with a neutralizing p40-specific antibody also ameliorated AD-associated pathology and cognitive deficits.

Several studies have demonstrated that microglia can express both neurotoxic and neurotrophic factors (65). Tan et al. hypothesized that the beneficial changes by treatment of SAMP8 mice with a neutralizing p40-specific antibody might be derived from a shift of microglia from an activated state with release of proinflammatory cytokines and inhibition of the clearance of Aβ toward an increased Aβ clearance and enhanced tissue remodeling.

Nevertheless, it cannot be excluded that the effector cells for p40 or IL-23 signaling are partly T cells, as described for other neuroinflammatory diseases. In a mouse models of AD, Aβ vaccination results in a reduction in amyloid burden concomitant with decreased expression of the IL-12Rβ1 receptor by T cells, the receptor subunit binding to p40 (66). Th17 cell-mediated neuroinflammation is involved in neurodegeneration of a rat AD model (67). Furthermore, activation of Th17 cells in AD patients has been demonstrated by Saresella et al. (68) and Th17 cells, which infiltrated into AD brain parenchyma, participate in neuroinflammation and neurodegeneration of AD by release of proinflammatory cytokines and by direct action on neurons *via* the Fas/FasL apoptotic pathway (67). The role of IL-23 and IL-17a, as the signature cytokine of Th17 cells, was also reviewed by Mohammadi Shahrokhi et al. (69), which identified IL-17a as a main inducer of neuroinflammation in AD. In contrast, Saksida et al. identified IL-17 as a rather protective factor. The central finding of the study was a lower production of IL-17 in gut-associated lymphoid tissue cells of aged 5xFAD mice probably due to impaired post-transcriptional stabilization of the IL-17 mRNA mediated by miR-155 (70). The decreased IL-17 level could impair the homeostasis of the immune system in the gut-associated lymphoid tissue, but could also contribute to inappropriate Aβ clearance in gut-associated lymphoid tissue and CNS. Another review speculates about the beneficial use of anti-IL-17A and anti-IL-23 antibody in AD by interfering with neutrophil infiltration and thereby suggets another possible effector cell in IL-23-mediated neuroinflammation in AD (71).

A shift from the Th17 cell/regulatory T cell balance favoring the proinflammatory Th17 side is suspected to contribute to exacerbation of autoimmune disorders (72). Since regulatory T cells delay disease progression in AD pathology (73), the role of regulatory T cells in IL-23 and AD should also be enlightened in further studies.

However, it is important to take into account that most data on the functional role of IL-23 in AD have been generated from mouse models, and in particular from the APP/PS1 model, which develops Aβ plaques by 6-8 months, but no tau pathology and does not cover all aspects of AD pathology (74). The effect of IL-23 especially on tau pathology is certainly worthwhile to investigate further.

Nevertheless, the current knowledge of the role of IL-23 and especially the IL-12/23 common subunit p40 are promising. The signaling pathways and effector cells involved in IL-23 and IL-12/23 p40 mediated immune response in AD should be enlightened in further studies, particularly in the clinical context.

### IL-23 an Attractive Therapeutic Target in AD?

Novel therapeutic options such as a variety of antibody therapies have led to significant progress in the treatment of many neurological diseases in recent years. However, the treatment of AD remains inadequate despite the immense progress in therapeutic options, so that the development of new therapeutic approaches remains a central aspect in AD research. Modulating the function of IL-23 appears to be an interesting target for AD although the precise signaling pathways and corresponding effector cells are not completely characterized. The beneficial results of the anti-IL-23 in preclinical studies could be transferable to the patient. While antibody therapies that interfere with the IL-23 are firmly established in the therapeutic concept for diseases such as psoriasis, spondylitis ankylosans, or inflammatory intestine illness (75–77), they are yet not established for the treatment of inflammatory CNS diseases. Administration of the p40 antibody ustekinumab was not successful in clinical trials in MS patients (78). Similarly, the anti-p40 antibody briakinumab showed only a slight benefit in terms of imaging progress and clinical relapse rate (79). Nevertheless, clinical trials blocking IL-23 and investigating whether it results in reduced neuroinflammation, reduced plaque burden, and improved cognitive impairment appear worthwhile. Considering the large number of patients already receiving an approved anti-IL23 therapy, studies of whether anti-IL23 therapy can prevent the development of AD would also be interesting.

These studies should be in particular feasible, as the yet approved anti-IL23 antibody therapies for autoimmune diseases showed a favorable risk profile in terms of safety of use, especially with regard to more severe infections or malignancies (75, 78–80).

## Discussion

Taken together, the currently available studies underline the impact of the proinflammatory cytokines of IL-23 and its subunit p40 in the pathogenesis of AD. In addition to clinical data showing the association of single nucleotide polymorphisms, IL-23 levels and AD, preclinical data demonstrate that IL-23 plays a crucial role in neuroinflammation, plaque formation in AD models and identify anti-IL-23 therapy as a promising new therapeutic approach. The data suggest that the aspects of IL-23 mediated neuroinflammation in AD remain an interesting research field and further data will enlighten the significance and signaling pathways of IL-23 in AD.

## Author Contributions

All authors prepared, corrected, and modified the manuscript.

## Conflict of Interest

The authors declare that the research was conducted in the absence of any commercial or financial relationships that could be construed as a potential conflict of interest.
